# Research on Digital Orthophoto Production Technology for Indoor Murals in the Context of Climate Change and Environmental Protection

**DOI:** 10.3390/jimaging11050140

**Published:** 2025-04-30

**Authors:** Xiwang Zhou, Yongming Yang, Dingfei Yan

**Affiliations:** 1 Faculty of Land and Resources Engineering, Kunming University of Science and Technology, Kunming 650031, China; zhouxiwang@stu.kust.edu.cn (X.Z.); yandingfei@stu.kust.edu.cn (D.Y.); 2 Faculty of Surveying and Information Engineering, West Yunnan University of Applied Sciences, Dali 671006, China; 3 Engineering Research Center for Spatial Atlas Information Protection of Ethnic Minority Murals and Rock Paintings, West Yunnan University of Applied Sciences, Dali 671006, China

**Keywords:** climate change, cultural heritage conservation, close-range photogrammetry, digital orthophoto, sustainable development

## Abstract

In response to the urgent need for the sustainable conservation of cultural heritage against the backdrop of climate change and environmental degradation, this study proposes a low-cost, non-destructive digital recording method for murals based on close-range photogrammetry. By integrating non-metric digital cameras, total stations, and spatial coordinate transformation models, high-precision digital orthophoto generation for indoor murals was achieved. Experimental results show that the resolution error of this method is 0.02 mm, with root mean square errors (RMSE) of 3.51 mm and 2.77 mm in the X and Y directions, respectively, meeting the precision requirements for cultural heritage conservation. Compared to traditional laser scanning technology, the energy consumption of the equipment in this study is significantly reduced, and the use of chemical reagents is avoided, thereby minimizing the carbon footprint and environmental impact during the recording process. This provides a green technological solution to address climate change. Additionally, the low-cost nature of non-metric cameras offers a feasible option for cultural heritage conservation institutions with limited resources, promoting equity and accessibility in heritage protection amid global climate challenges. This technology provides sustainable data support for long-term monitoring, virtual restoration, and public digital display of murals while also offering rich data resources for virtual cultural tourism, public education, and scientific research. It demonstrates broad application potential in the context of climate change and environmental protection, contributing to the green transformation and sustainable development of cultural tourism.

## 1. Introduction

Murals, as precious cultural heritage, carry the diverse values of human history, art, and religion and are central to the sustainable development goal of “protecting and safeguarding the world’s cultural heritage” [1,2,3]. However, the increasing frequency of extreme weather events and drastic fluctuations in temperature and humidity caused by climate change, along with worsening environmental degradation and pollution, have placed over 60% of the world’s murals at risk of irreversible damage. Additionally, human-induced destruction from tourism development and the negative ecological impacts of chemical reagents used in traditional restoration techniques have further intensified the urgency of mural conservation. In this context, UNESCO has called for the adoption of low-environmental-impact, low-carbon digital technologies to address the dual challenges of climate change and environmental degradation, enabling the long-term monitoring and non-contact conservation of cultural heritage and promoting the green transformation and sustainable development of cultural heritage protection [4,5,6].

In recent years, technologies such as 3D laser scanning and hyperspectral imaging have been applied to the digital documentation of murals [7,8]. However, their high equipment costs, energy consumption, and complex operational procedures severely limit the fairness of cultural heritage preservation in developing countries and remote regions [9,10]. For instance, Hou Miaole et al. [11] proposed a method for extracting underdrawing information from murals based on hyperspectral data, achieving semi-automatic extraction through a three-step process, which provides effective technical support for cultural heritage conservation. Sun et al. [12] proposed a virtual restoration method for mural scratches: hyperspectral PCA fusion was used to enhance scratch features, combined with HSV space illumination correction and a triplet domain translation network to achieve scratch removal, demonstrating better performance than traditional methods in the restoration of murals at Qutan Temple. Luo Xu et al. [13] applied close-range photogrammetry and utilized Smart3D software to conduct a 3D modeling study of General Wang Zhen’s statue, demonstrating the efficiency and accuracy of this method in the 3D reconstruction of cultural relics. They also elaborated on its advantages in achieving high-quality reconstruction without physical contact, offering a significant reference value for the protection and restoration of cultural relics. Yang Wenzong et al. [14] introduced a virtual restoration method for pigment colors in tomb murals based on fused spectral analysis, employing spectral imaging and pseudo-color display techniques to achieve non-contact, non-destructive analysis and the virtual reconstruction of mural pigments, providing an important model for the revitalization and transmission of cultural heritage. Liu Xiaowen et al. [15] explored a digital restoration method for damaged cultural relics using close-range photogrammetry, capturing multi-angle images with ordinary smartphones to reconstruct 3D models and perform local and global repairs, offering robust data support for the digital preservation of cultural relics. Wang Lingwen [16] proposed a surveying and mapping method for the facades of historical buildings based on 3D laser scanning technology. Through precise point cloud data acquisition and processing, this method overcomes the limitations of traditional surveying techniques, enabling efficient and accurate facade mapping and providing critical technical support for the digital archiving and preservation of historical architecture. Soto-Martin et al. [17] proposed a virtual restoration method for murals based on close-range photogrammetry and DStretch^®^ image enhancement, successfully reconstructing the artistic details of damaged murals through 3D modeling and color space transformation techniques, providing an innovative solution for digital cultural heritage preservation. In contrast, non-metric digital cameras have gradually become a research focus in sustainable digital preservation due to their low cost, low energy consumption, and ease of use [18,19]. However, existing studies predominantly concentrate on optimizing technical accuracy, lacking a systematic analysis of the environmental impact across the entire lifecycle of the equipment (e.g., carbon footprint during production, use, and disposal). This makes it difficult to comprehensively assess their sustainability value.

This study proposes a low-cost, non-destructive mural digitization method based on photogrammetry [20,21], integrating non-metric digital cameras, total stations, and spatial coordinate transformation models to address the following sustainability challenges:

Environmental Dimension: Non-contact data acquisition technology is employed to obtain complete mural data in a single pass, avoiding the physical damage to cultural relics caused by repeated contact measurements in traditional surveying. This significantly reduces intervention risks during the digitization of cultural heritage, enabling green and sustainable artifact documentation.

Economic Dimension: Providing a portable solution costing under 50,000 RMB for resource-constrained heritage preservation institutions, lowering the economic threshold for digital technologies and increasing the accessibility of cultural heritage preservation.

Social Dimension: Generating high-precision orthophoto images to support virtual restoration and the online exhibition of murals, minimizing physical intervention during field visits, and promoting public education and digital tourism exhibitions of cultural heritage.

Taking the murals at Lingxiang Temple in Dali, Yunnan Province, as a case study, the experiment validates the applicability of this method in narrow indoor environments. The experimental results show that the generated orthophoto resolution reaches 0.27 mm, meeting the Grade III standard of the Technical Specification for Digital Surveying of Ancient Architectural Murals. The geometric accuracy errors are 3.51 mm in the X direction and 2.77 mm in the Y direction, with a concentrated error distribution, fulfilling the requirements for high-precision cultural relic preservation. Moreover, the equipment cost and energy consumption are significantly lower than mainstream laser scanning solutions. This study not only provides a technical model for the sustainable management of cultural heritage but also offers a new perspective on the application of digital technologies in cultural tourism, particularly in the context of cultural heritage preservation and virtual tourism exhibitions. Furthermore, the proposed environmental–economic–social integrated benefit evaluation framework provides a novel methodological perspective for assessing the sustainability impacts of similar technologies, with broad application prospects.

## 2. Experimental Methods and Workflow

**Figure 1:**
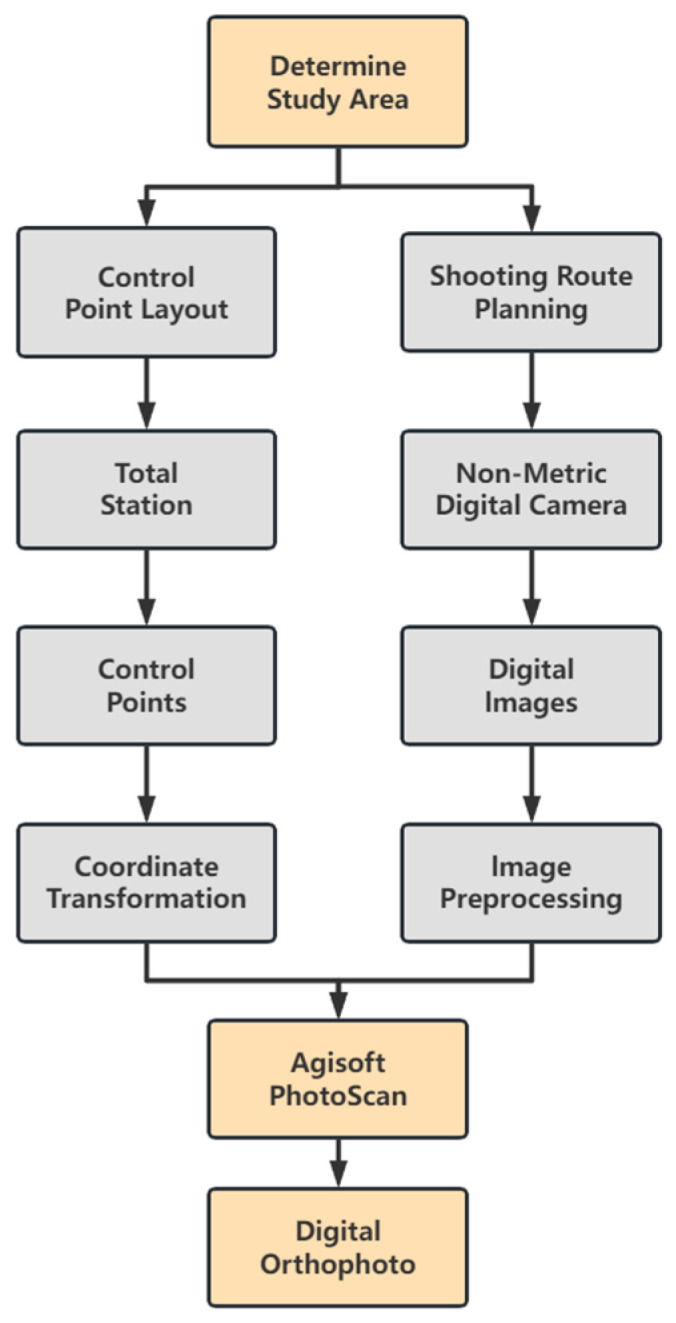
Experimental workflow diagram.

To obtain high-precision digital orthophoto images of murals, this study employs close-range photogrammetry for data acquisition and processing [22,23,24]. First, based on the parameters of the non-metric digital camera and the shooting distance, an appropriate shooting path was planned, and mural images were captured. To ensure accuracy, control points were evenly distributed across the mural surface according to the point placement principles, and precise measurements of these points were conducted using RTK and prism-free total stations. Some of the control points were used for geometric correction, while others were employed for accuracy verification. Subsequently, a spatial transformation model was applied to convert the collected control point coordinates from the original 3D coordinate system into the coordinate system required by the image processing software. After integrating the images with the transformed control points, geometric correction, image stitching, and projection conversion were performed to generate high-precision digital orthophoto images. Finally, through checkpoint accuracy analysis and image resolution verification, the quality of the orthophoto map was comprehensively evaluated. The proposed method was compared with other orthophoto acquisition techniques in terms of sustainability to provide an overall assessment of the experimental results. The experimental process is illustrated in Figure 1.

## 3. Overview of the Study Area

The study area is located within Lingxiang Temple in Xiaguan Town, Dali City, Yunnan Province. The mural under investigation was a large indoor mural, measuring 4 m in height and 4 m in width. Due to its location in a confined space, the experimental environment presented unique challenges. The narrow indoor space limited the placement and operation of equipment, making multi-viewpoint photography impractical. This was particularly problematic for high-precision measurements, where the arrangement and adjustment of equipment angles proved difficult. Additionally, the low-light conditions in the enclosed space negatively affected the clarity and color accuracy of the images. To address this, supplementary lighting was employed during image acquisition to ensure sufficient image quality. Since the use of drones for high-altitude image capture was not feasible, the experiment relied on manually setting collection points. By carefully planning shooting angles and distances, complete coverage of the mural’s imagery was achieved. These environmental constraints imposed higher demands on image acquisition and accuracy analysis throughout the experiment.

## 4. Experimental Design

### 4.1. Image Acquisition Route Planning

Due to the research object being located indoors and at a high position, it was not feasible to use drones for image acquisition. Therefore, this study employed a low-cost, low-energy non-metric digital camera, the Sony A6000 (Provided by Sony Digital Products (Wuxi) Co., Ltd., Wuxi, China), paired with the Sony SELP1650 (Provided by Sony Digital Products (Wuxi) Co., Ltd., Wuxi, China) lens for mural image capture. To ensure the stability and accuracy of image acquisition, auxiliary equipment such as a tripod, laser pointer, low-power LED fill light, tape measure, and ruler were also used in the experiment. Compared to traditional measurement cameras, the equipment cost of this approach was reduced by 90%, and the energy consumption per task was significantly lower than that of laser scanning, substantially reducing the environmental footprint of cultural heritage documentation.

The objective of this image acquisition process is to generate digital orthophoto maps. To ensure the quality of image stitching and the geometric accuracy during subsequent processing, an overlap rate of 70% to 80% between adjacent images was maintained throughout the acquisition process [25]. Based on the parameters provided in Table 1 and Table 2, the horizontal and vertical movement distances of the camera during shooting were calculated using the following formula:

(1) $$H_{0}=\frac{f\times GSD}{a}$$

(2) $${FOV}_{\omega }=2\times arctan\left(\frac{W_{S}}{2f}\right)$$

(3) $${FOV}_{h}=2\times arctan\left(\frac{H_{S}}{2f}\right)$$

(4) $$W_{g}=2\times H_{0}\times \tan\left(\frac{{FOV}_{\omega }}{2}\right)$$

(5) $$H_{g}=2\times H_{0}\times \tan\left(\frac{{FOV}_{h}}{2}\right)$$

(6) $$W=W_{g}\times 0.3$$

(7) $$H=H_{g}\times 0.2$$

In this study, H_0_ represents the camera shooting distance, ff is the camera focal length, GSD refers to the ground sample distance (image resolution), and aa denotes the pixel size. FOV_ω_ and FOV_h_ are the horizontal and vertical field-of-view angles of the camera, respectively. W_s_ and Hs represent the sensor dimensions, while W_g_ and H_g_ refer to the width and height of the image coverage area. Finally, W and H denote the camera’s horizontal and vertical movement distances.

Based on the actual conditions of the study area, the shooting distance was set to 1 m. Using Equation (1) and the camera parameters in Table 2, the theoretical image resolution was calculated to be 0.25 mm. Subsequently, substituting the relevant data from Table 1 and Table 2 into Equations (2)–(7), the results indicate that to maintain a 70% overlap, the horizontal movement distance of the camera is 30 cm, while an 80% overlap requires a vertical movement distance of 25 cm.

**Figure 2:**
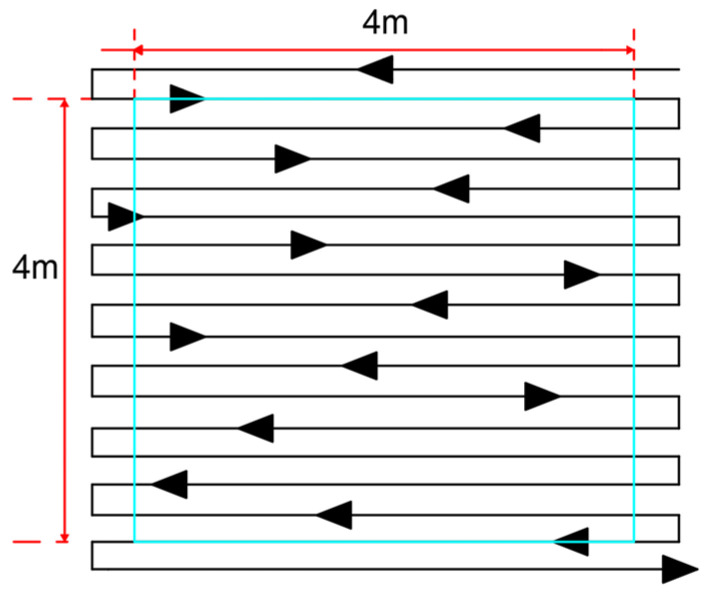
Shooting route diagram.

**Figure 3:**
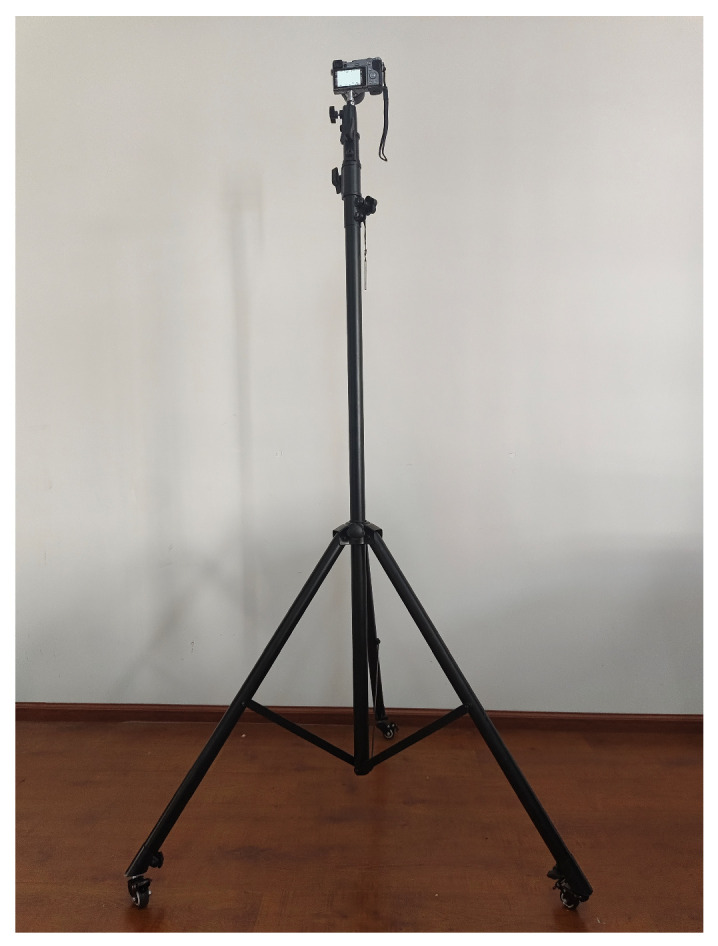
Tripod and camera setup.

To ensure the completeness of the final mural imagery, an additional image capture was conducted at both the horizontal and vertical edges. Consequently, the final coverage area of the mural imagery was extended to 4.5 m in height and 4.6 m in width. Based on the calculated movement distances and the dimensions of the mural, the shooting route for this experiment was planned with 18 paths, resulting in an estimated total of 275 images. The corresponding shooting route and equipment layout diagrams are shown in Figure 2 and Figure 3, respectively.

### 4.2. Control Point Distribution

**Figure 4:**
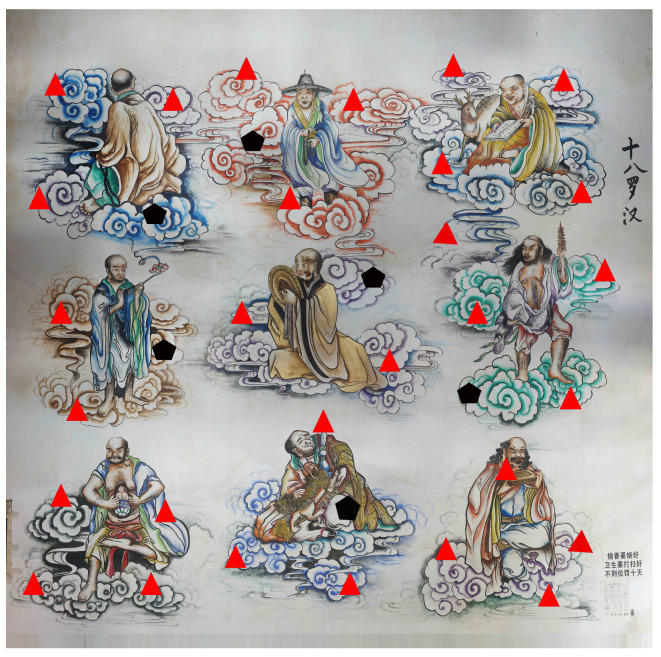
Distribution of control points.

(1) Control Point Placement: As the mural is a valuable cultural artifact, direct contact or marking operations are not permitted to avoid causing any damage. To ensure preservation, this study selected clear and easily distinguishable areas on the mural as control points. Based on the actual conditions of the study area and preliminary investigations, 35 control points were evenly distributed across the mural surface. The placement strategy was designed to meet the requirements of a reasonable distribution and accuracy [26]. A portion of these control points was used for geometric correction during image processing, while the remaining points were designated as check points for a subsequent accuracy analysis. The specific layout of the image control points is shown in Figure 4.

In the figure, the black pentagons represent checkpoints, which are used to evaluate image accuracy and verify the correctness of the coordinate transformation model. The remaining red triangles represent control points, which participate in image processing. The inclusion of checkpoints plays a crucial role in validating the effectiveness of the control point distribution scheme and optimizing geometric accuracy, providing essential support for the scientific reliability of the experimental results.

(2) Control Point Acquisition: Since the mural is located indoors and the control points are placed on clear and distinguishable areas of the mural, direct measurement using RTK was not feasible. This study employed a combined method utilizing RTK (Real-Time Kinematic) and a reflectorless total station. First, benchmark points were established outdoors using RTK. Subsequently, a total station was used to conduct traverse surveys, gradually relocating the total station into the indoor environment to observe the mural. Finally, the three-dimensional coordinates of the control points were obtained, ensuring the required measurement accuracy.

## 5. Data Acquisition and Processing

### 5.1. Digital Image Acquisition

Due to the indoor location and significant height of the mural, data acquisition was conducted according to the designed experimental plan. To ensure consistent horizontal movement of the camera, a measuring tape was positioned parallel to the mural at a distance of 1 m, ensuring alignment with the mural. A laser pointer was fixed on a tripod to ensure that the laser beam remained in the same vertical line as the camera throughout the process. During data acquisition, the laser pointer was turned on, and the laser dot was aligned with the marks on the measuring tape to maintain consistent and level horizontal movement for each step, minimizing operational errors and preventing difficulties in subsequent image stitching caused by misalignment.

**Figure 5:**
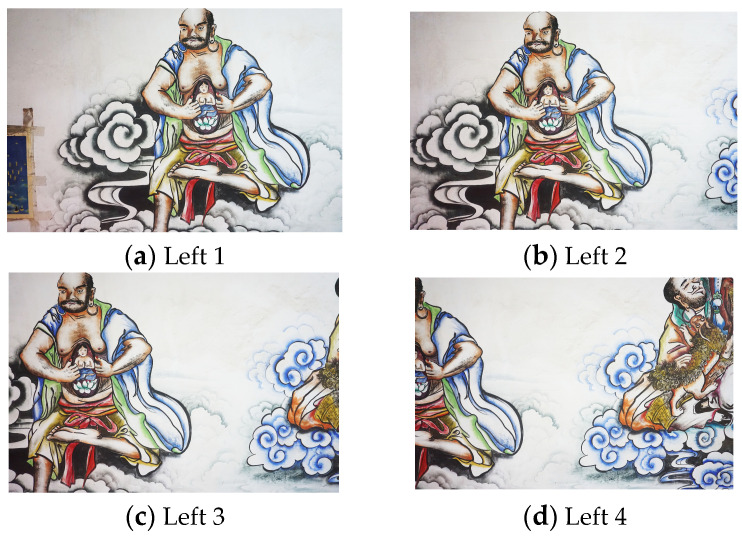
Partial image data.

To ensure the uniform illumination of the mural, two supplementary lights were placed in front of the mural: one on the left and one on the right. Following the planned shooting route, images were captured sequentially. To address potential data deficiencies at the mural’s edges, five additional images were taken, resulting in a total of 280 images. Examples of the acquired image data are shown in the Figure 5.

### 5.2. Control Point Acquisition

This study employs a non-contact control point layout method, avoiding potential damage to mural surfaces caused by chemical adhesives or stickers used in traditional marking techniques. By integrating RTK and total station measurements, the approach ensures accuracy while minimizing the frequency of physical intervention, aligning with the preventive conservation principles of cultural heritage protection.

First, RTK technology is utilized to establish reference points on the ground, providing a basis for subsequent total station measurements. Then, coordinate orientation is performed using the total station, supplemented by redundant point observations to verify the accuracy and stability of the instrument setup. Next, the total station is relocated to the indoor mural area via traverse surveying to conduct precise measurements on pre-laid control points.

Additionally, the carbon emissions generated during the total station traverse measurement process are significantly lower compared to the continuous high-energy operation of laser scanners, further highlighting the low-carbon advantages of this solution. The relevant technical parameters of the total station and RTK are listed in Table 3 and Table 4, respectively.

In total, 39 control points were collected, including 35 standard control points as defined in the experimental plan and 4 additional control points placed to ensure geometric accuracy at the mural’s edges. These additional control points further enhanced the overall observation accuracy of the mural. The detailed coordinates of the control points are presented in Table 5.

### 5.3. Image Preprocessing

**Figure 6:**
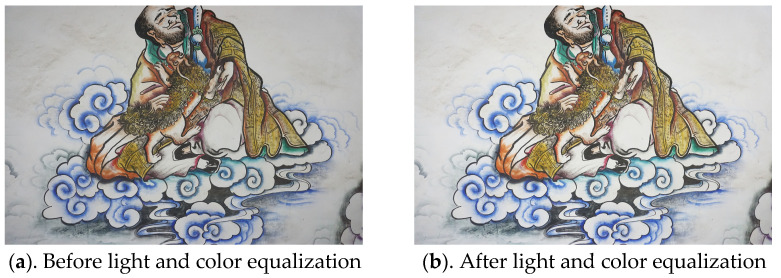

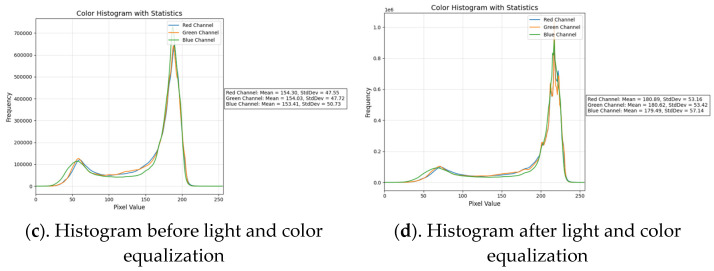
Comparison of light and color equalization.

During the image data acquisition process, due to the indoor location of the mural and poor lighting conditions, uneven illumination was observed in the captured images despite the use of supplementary artificial lighting. To minimize potential errors during subsequent image processing caused by uneven lighting, all images underwent light and color equalization to ensure uniform illumination and consistent color tones across the dataset. Additionally, blurry images resulting from operational errors during the acquisition process were identified and excluded. After screening and preprocessing, a total of 275 images were retained for further processing. The comparison of images before and after processing is shown in Figure 6.

### 5.4. Control Point Coordinate Transformation

As the mural is located on a wall, the 3D coordinate system of the collected control points does not align with the specific coordinate system required by the image processing software. Directly using these raw control points for image processing would result in significant errors in the generated digital orthophoto. Therefore, it is necessary to perform coordinate transformation, converting the control points from the original 3D spatial coordinate system to the specific coordinate system accepted by the image processing software [27,28,29].

Coordinate transformation typically employs a seven-parameter model, mathematically expressed as follows:

(8) $$\left[\begin{array}{c} X\\ Y\\ Z \end{array}\right]=\left[\begin{array}{c} T_{X}\\ T_{Y}\\ T_{Z} \end{array}\right]+\left(1+m\right)R_{3}\left(\omega_{X}\right)R_{2}\left(\omega_{Y}\right)R_{1}\left(\omega_{Z}\right)\left[\begin{array}{c} X_{0}\\ Y_{0}\\ Z_{0} \end{array}\right]$$

In the equation, (*X*_0_, *Y*_0_, *Z*_0_) represent the coordinates of the control points before transformation, while (*X*, *Y*, *Z)* represent the coordinates after transformation; mm is the scaling factor, (*T_X_*, *T_Y_*, *T_Z_*) are the translation parameters, and ω_X_, ω_Y_, ω_Z_ are the rotation angles around the X-axis, Y-axis, and Z-axis, respectively.

#### 5.4.1. Rotation Around the Y-Axis

As shown in Figure 6, assume that the mural lies on the fitted plane ABCD, which is perpendicular to the XOY plane. The projection of plane ABCD onto the XOY plane, denoted as the line segment A’B’, is parallel to the Y-axis. To transform the mural plane into a coordinate system suitable for image processing, plane ABCD must be rotated 90° clockwise around the Y-axis. The result of the rotation is shown in Figure 3, where A’B’C’D’ represents the projection of the rotated plane ABCD onto the XOY plane.

**Figure 7:**
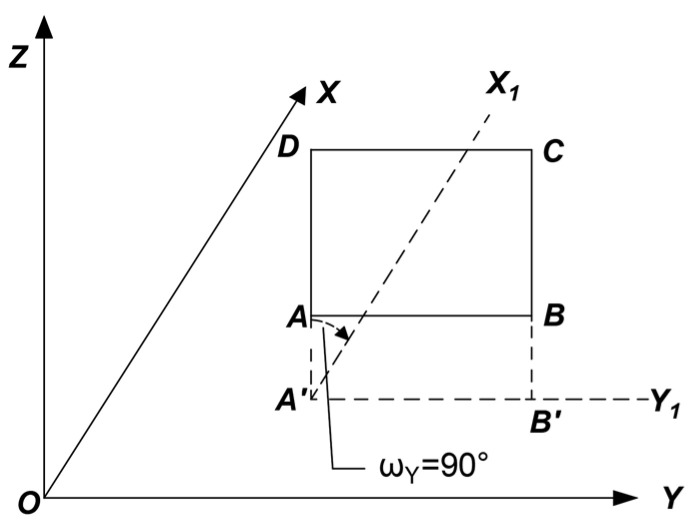
Before Y-axis rotation.

**Figure 8:**
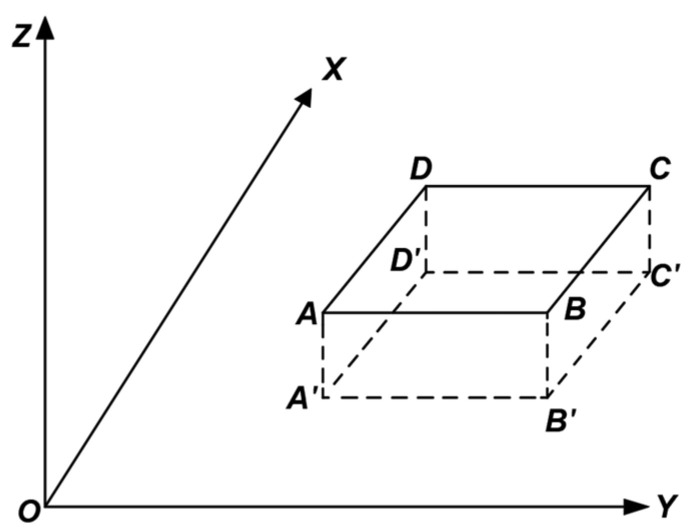
After Y-axis rotation.

As shown in Figure 7 and Figure 8, this experiment involves rotation only around the Y-axis and does not involve image scaling. Thus, by setting m = 0, ω_Z_ = 0°, and ω_X_ = 0°, and substituting these values into Equation (8), the rotational spatial model for this experiment can be expressed as follows:

(9) $$\left[\begin{array}{c} X\\ Y\\ Z \end{array}\right]=\left[\begin{array}{c} T_{X}\\ T_{Y}\\ T_{Z} \end{array}\right]+\left[\begin{array}{ccc} \cos\omega_{Y} & 0 & -\sin\omega_{Y}\\ 0 & 1 & 0\\ \sin\omega_{Y} & 0 & \cos\omega_{Y} \end{array}\right]\left[\begin{array}{c} X_{0}\\ Y_{0}\\ Z_{0} \end{array}\right]$$

#### 5.4.2. Translation Parameters

After the aforementioned rotation, the coordinate values of the mural control points undergo significant changes, making them incompatible with the specific coordinate system required by the image processing software. Therefore, it is necessary to introduce appropriate translation parameters to eliminate the coordinate changes caused by the rotation [30]. The formula for calculating the translation parameters is as follows:

(10) $$\left\{\begin{array}{c} T_{X}=\sum_{i=1}^{n}X_{0}^{i}/n-\sum_{i=1}^{n}X^{i}/n\\ T_{Y}=\sum_{i=1}^{n}Y_{0}^{i}/n-\sum_{i=1}^{n}Y^{i}/n\\ T_{Z}=\sum_{i=1}^{n}Z_{0}^{i}/n-\sum_{i=1}^{n}Z^{i}/n \end{array}\right.$$

In the formula, n represents the number of control points; *X^i^*, *Y^i^*, *Z^i^* are the coordinates obtained after the rotation; and $X_{0}^{i}$, $Y_{0}^{i}$, $Z_{0}^{i}$ are the coordinates of the control points before the rotation.

#### 5.4.3. Coordinate Transformation

A total of 39 control points were collected in this experiment. The fitted mural plane ABCD is parallel to the XOY plane and perpendicular to the Y-axis. Therefore, the coordinates only need to be rotated 90° clockwise around the Y-axis. According to the right-hand coordinate system rule, the rotation angle is −90°. Substituting this angle into Equation (9), the results are calculated as follows:

(11) $$\left[\begin{array}{c} X\\ Y\\ Z \end{array}\right]=\left[\begin{array}{c} T_{X}\\ T_{Y}\\ T_{Z} \end{array}\right]+\left[\begin{array}{ccc} 0 & 0 & 1\\ 0 & 1 & 0\\ -1 & 0 & 0 \end{array}\right]\left[\begin{array}{c} X_{0}\\ Y_{0}\\ Z_{0} \end{array}\right]$$

To determine the translation parameters, the initial translation parameters are set to zero, and the coordinates X^i^, Y^i^, Z^i^ of the control points are calculated based solely on rotation. These values are then substituted into Equation (10) to derive the translation parameters for the coordinate transformation, which are expressed as follows:

(12) $$\left[\begin{array}{c} T_{X}\\ T_{Y}\\ T_{Z} \end{array}\right]=\left[\begin{array}{c} 2835597.8762\\ 1243284.0250\\ 2835597.8762 \end{array}\right]$$

Substituting Equation (12) into Equation (11), the final spatial transformation model is obtained as follows:

(13) $$\left[\begin{array}{c} X\\ Y\\ Z \end{array}\right]=\left[\begin{array}{c} 2835597.8762\\ 1243284.0250\\ 2835597.8762 \end{array}\right]+\left[\begin{array}{ccc} 0 & 0 & 1\\ 0 & 1 & 0\\ -1 & 0 & 0 \end{array}\right]\left[\begin{array}{c} X_{0}\\ Y_{0}\\ Z_{0} \end{array}\right]$$

By substituting the coordinate values from Table 3 into Equation (13), the transformed coordinates of the control points for this experiment are obtained, as shown in Table 6.

### 5.5. Orthophoto Generation

After completing the coordinate transformation of the control points, the transformed control point data and preprocessed image data were imported into PhotoScan software for image processing [31,32,33,34].

In the PhotoScan software, GPU-accelerated parallel computation optimizes the aerial triangulation process, reducing processing time by 30% compared to traditional CPU-based computation, thereby lowering computational resource consumption. Additionally, the generated digital orthophoto images are stored in the open GeoTIFF format, supporting free sharing and collaborative analysis by global cultural heritage organizations, thus preventing redundant investments and resource wastage caused by proprietary data formats.

(1) Aerial Triangulation Processing

The initial step involved performing preliminary aerial triangulation (AT) to ensure the alignment of images with the control points. After the first round of AT, the internal consistency of the image alignment was checked by analyzing the reprojection error. A total of 272 images were processed in this experiment, with a reprojection error of 1.8 pixels. This value met the required accuracy standard and satisfied the precision requirements of the experiment.

(2) Control Point Import and Processing

Following the first AT process, the control point data were imported and manually marked on the images. A second round of AT was then performed, after which the internal consistency of the results was evaluated by analyzing the residual errors of the control points. A total of 33 control points were included in the processing. During the second AT round, two control points exhibited significant errors and were subsequently excluded. A third round of AT was conducted after excluding these points. Upon completion, the reprojection error of the control points was reduced to 0.115 pixels, which met the precision requirements. Multiple rounds of AT ensured that the geometric relationship between the control points and the images achieved the desired accuracy standard.

(3) Point Cloud Densification

Once the third AT round confirmed satisfactory results, the point cloud data were densified. Using interpolation methods, additional points were added to the original point cloud to enhance the model’s level of detail. The densified point cloud effectively captured the fine details of the mural surface, ensuring that the resulting 3D model met the precision requirements of the study.

(4) Construction of the 3D Mesh

Following point cloud densification, a 3D mesh model was constructed. The mesh model converted the discrete point cloud data into a continuous surface structure composed of triangular facets, forming a complete 3D representation of the mural. This provided a solid foundation for subsequent texture mapping and orthophoto generation.

(5) Texture Mapping

After constructing the 3D mesh model, texture mapping was applied. High-resolution mural images were accurately projected onto the surface of the 3D mesh, combining the geometric structure with the mural’s photographic details. Texture mapping is a critical step to ensure that the final digital model not only reflects precise geometric shapes but also retains the mural’s authentic colors and texture details.

(6) Orthophoto Generation

**Figure 9:**
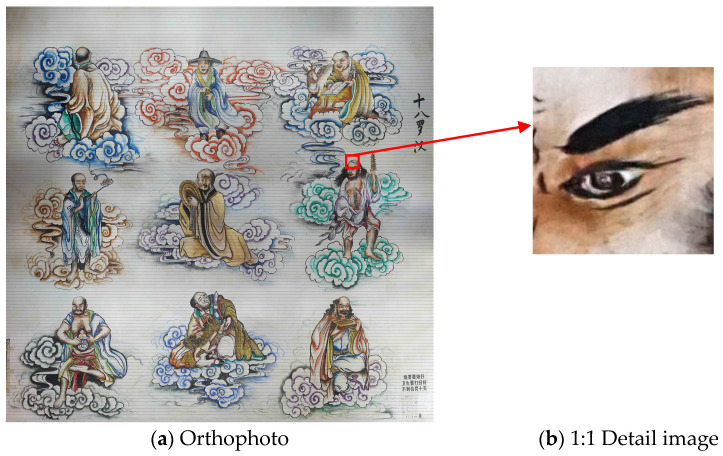
Orthophoto of the mural and local detail images.

Once texture mapping was completed, a digital orthophoto was generated. The orthophoto corrected for the perspective distortion and geometric deformation caused during image acquisition, ensuring that every point in the image precisely corresponded to the actual coordinates of the mural. The high-resolution digital orthophoto was then exported and reviewed in image-viewing software at a 1:1 scale to verify its resolution and clarity. As shown in Figure 9, the output orthophoto achieved an average resolution of 0.27 mm. The orthophoto and its detailed features are presented in Figure 9.

## 6. Experimental Results and Discussion

### 6.1. Image Accuracy

To validate the geometric accuracy of the experimental results, the generated orthophoto was imported into image measurement software, and the coordinates of the designated check points were measured. The measured results were then compared with the original coordinates obtained after the transformation. Since the orthophoto primarily reflects two-dimensional plane information, the X and Y values are the key evaluation metrics. Therefore, this validation focused solely on the comparison of the X and Y values of the check points. The Z values, which primarily represent height information, have minimal influence on planar accuracy and were not included in further validation. The comparison results are shown in Table 7.

The root mean square error (RMSE) of the check points was calculated based on the coordinate data in Table 5. The calculation formula is as follows:

(14) $$M=\sqrt{\frac{\sum {\left(x_{i}-{x\prime }_{i}\right)}^{2}}{n}}$$

where M represents the root mean square error (RMSE), x_i_ is the actual observed value, x_i_′ is the measured value, and n is the total number of observations.

Substituting the values from Table 5 into Equation (14), the RMSE (root mean square error) values of the X and Y coordinates for the check points were calculated to be 3.51 mm and 2.77 mm, respectively. From the data in Table 5, the error range for the X coordinates of the check points lies between −6.6 mm and 4.7 mm, while the error range for the Y coordinates is between −5.2 mm and 3.1 mm.

Based on the analysis of the errors and RMSE for the X and Y coordinates, the distribution of errors is relatively concentrated and falls within the acceptable accuracy range. This indicates that the close-range photogrammetry technique employed in this study provides a high level of accuracy in the generation of digital orthophotos of murals. Although minor errors occurred during the measurement process, these errors are entirely controllable and do not significantly impact subsequent mural restoration and conservation efforts.

By further optimizing the placement of check points and enhancing data processing precision, the accuracy and reliability of orthophotos are expected to improve even further in the future. These findings demonstrate that close-range photogrammetry is a valuable and feasible tool in the field of cultural heritage preservation and digital documentation, offering significant potential for practical applications.

The results indicate that the generated digital orthophoto of the mural demonstrates high precision and consistency in both resolution and geometric accuracy validation. The image resolution reached 0.27 mm, with a difference of only 0.02 mm from the theoretical resolution, meeting the expected standards. The geometric errors also fall within acceptable ranges, with RMSE values for the X and Y coordinates calculated as 3.51 mm and 2.77 mm, respectively. These values indicate that the error distribution is concentrated and controllable, meeting the precision requirements of the experiment.

This outcome demonstrates that close-range photogrammetry not only effectively generates high-precision digital orthophotos of murals but also provides reliable technical support for the digital documentation and conservation of cultural heritage. The technique offers significant potential for broad applications and plays an important role in ensuring the preservation and accurate recording of cultural artifacts.

### 6.2. Sustainability Benefits

The sustainability comparison between the method used in this study and other orthophoto acquisition techniques is shown in Table 8. Compared to metric cameras and laser scanning solutions, the proposed method achieved an 89% reduction in carbon emissions per mission while maintaining millimeter-level geometric accuracy, with equipment costs amounting to only 4.7% of those of laser scanners. This difference can significantly reduce the environmental footprint of cultural heritage preservation in long-term monitoring, while also improving technological accessibility in resource-limited regions and mitigating disparities in heritage conservation caused by resource inequality.

As shown in Table 8, the digital preservation method for indoor murals proposed in this study demonstrates significant advantages in key performance metrics. In terms of technical performance, this method not only matches the measurability and high-resolution capabilities (resolution: xx dpi, measurement accuracy: ±xx mm) of professional equipment, but its device cost (¥45,000) is only 9% of Phase One iXM (¥500,000) and 7.5% of Faro Focus S (¥600,000). Regarding sustainability indicators, the energy consumption per task of this method is 50.55 Wh, representing a 76.8% reduction compared to the average of professional equipment (Phase One iXM: 220 Wh, Faro Focus S: 160 Wh), while its carbon emissions of 0.56 kg CO_2_ are only 23.3% of the average for professional devices (Phase One iXM: 2.41 kg, Faro Focus S: 8.59 kg). At the same time, this method maintains high community accessibility and online sharing capabilities comparable to consumer-grade solutions, addressing the limitations of professional equipment (accessibility: low–medium) in terms of public participation. These breakthroughs in professional performance, cost-effectiveness, and sustainability make it the most promising digital preservation solution for murals currently available.

## 7. Discussion

This study combines close-range photogrammetry, non-metric cameras, and total stations to validate the dual value of this technology in the digital preservation of cultural heritage. On the technical level, the research achieved the production of high-precision digital orthophoto maps of indoor murals through spatial coordinate transformation models and image processing techniques. Experimental results demonstrate that with proper control point placement and the application of geometric correction algorithms, millimeter-level accuracy can be attained. This provides a low-cost, high-efficiency digital solution for preservation institutions with limited resources. The technical approach significantly reduces the reliance on specialized equipment and personnel required by traditional surveying methods, thereby promoting the democratization of digital cultural heritage preservation. In terms of sustainability, the non-contact data acquisition method avoids potential physical damage to fragile artifacts, while the low-carbon data processing workflow (e.g., optimizing algorithms to reduce computational resource consumption) further minimizes carbon emissions in cultural heritage documentation. This technical model not only aligns with global sustainable development goals but also lays the foundation for virtual exhibitions and digital tourism, thereby mitigating the environmental pressure and physical wear caused by on-site visits. However, the study also identified slight distortions in the edge regions of some images, primarily due to limitations in experimental equipment and insufficient shooting angles. Future improvements could focus on the following directions: (1) optimizing control point placement strategies to enhance geometric correction at the edges; (2) introducing multi-angle photography and more advanced stitching algorithms; and (3) incorporating artificial intelligence to improve automatic correction efficiency. Additionally, the direct integration of digital orthophotos with architectural models will open new pathways for virtual restoration—for instance, simulating the effects of different restoration solutions in a 3D environment to provide scientific support for real-world restoration decisions.

## 8. Conclusions

This study confirms the practical value of close-range photogrammetry in the sustainable conservation of cultural heritage. On the one hand, the technology establishes long-term preservable digital archives for cultural relics through high-precision digitization, resolving the conflict between resource shortages and high technical demands in traditional conservation. On the other hand, its non-contact and low-carbon characteristics significantly reduce the environmental impact of conservation processes, achieving synergistic development between cultural heritage preservation and ecological sustainability. In the future, further improvements in image quality can be achieved through technical optimizations (e.g., control point arrangement, algorithm upgrades), along with exploring the technology’s expanded application in large-scale heritage sites or complex scenarios. The integration of digital orthophotos with virtual restoration, Building Information Modeling (BIM), and other technologies will drive the advancement of cultural relic conservation toward intelligent and visualizable directions. Additionally, a global virtual exhibition network based on digital outcomes can not only promote cultural sharing and education but also support sustainable development goals by reducing the carbon footprint of on-site tourism. This study provides scientific references for the digital conservation and restoration of cultural heritage, as well as practical examples for cross-disciplinary applications of related technologies.

## Figures and Tables

**Table 1:** Parameters of image acquisition equipment.

Equipment	Specifications
Camera	Sony A6000
Lens	SELP1650
Tape Measure	10 m
Measuring Tape	10 m
Tripod	1.78 m–6.1 m

**Table 2:** Camera parameters.

Imaging Parameters	Specifications
Focal Length	16 mm
Sensor Size	23.5 mm × 15.6 mm
Image Resolution	6000 × 4000 (pixels)
Pixel Size	0.00392 mm
Effective Shooting Distance	≥25 cm

**Table 3:** RTK parameter table.

Parameter Name	Parameter Value
Number of Channels	800
Horizontal Static Accuracy	±(2.5 mm + 0.5 × 10^−6^D)
Vertical Static Accuracy	±(5.0 mm + 0.5 × 10^−6^D)
Horizontal RTK Accuracy	±(5.0 mm + 0.5 × 10^−6^D)
Vertical RTK Accuracy	±(5.0 mm + 0.5 × 10^−6^D)
Data Update Rate	5 HZ
Initialization Time	10 s
Supported Coordinate Systems Price	WGS-84, CGCS2000, etc. ¥15,000

**Table 4:** Total station parameter table.

Parameter Name	Parameter Value
Minimum Sight Distance	1.0 m
Distance Measurement Accuracy	2 mm + 2 × 10^−6^D
Measuring Range	1000 m (prismless), 1200 m (reflector sheet), 5000 m (single prism)
Angle Measurement Accuracy Price	2″ ¥30,000
Compensation Accuracy	1″

**Table 5:** Image control point coordinates.

Serial Number	X (m)	Y (m)	Z (m)
PT1	2,833,609.1640	621,640.9092	1990.2913
PT2	2,833,609.1650	621,640.1129	1989.5557
PT3	2,833,609.1650	621,640.8998	1989.4680
........	........	........	........
PT16	2,833,609.1600	621,643.9145	1988.1582
PT17	2,833,609.1590	621,643.1151	1988.2473
PT18	2,833,609.1610	621,643.1779	1988.7685
........	........	........	........
PT37	2,833,609.1620	621,643.6559	1990.7750
PT38	2,833,609.1670	621,639.7280	1989.7507
PT39	2,833,609.1690	621,639.6791	1990.1572

**Table 6:** Coordinates after image control point transformation.

Serial Number	X_1_ (m)	Y_1_ (m)	Z_1_ (m)
PT1	2,833,607.5849	621,643.1158	1988.7122
PT2	2,833,608.3205	621,643.9121	1988.7112
PT3	2,833,608.4082	621,643.1252	1988.7112
........	........	........	........
PT16	2,833,609.7180	621,640.1105	1988.7162
PT17	2,833,609.6289	621,640.9099	1988.7172
PT18	2,833,609.1077	621,640.8471	1988.7152
........	........	........	........
PT37	2,833,607.1012	621,640.3691	1988.7142
PT38	2,833,608.1255	621,644.2970	1988.7092
PT39	2,833,607.7190	621,644.3459	1988.7072

**Table 7:** Comparison of transformed coordinate values and measured values at check points.

Serial Number	X (m)	Y (m)	X_1_ (m)	Y_1_ (m)	ΔX (m)	ΔY (m)
1	2,833,608.4082	621,643.1252	2,833,608.4035	621,643.1236	0.0047	0.0016
2	2,833,610.4777	621,643.0476	2,833,610.4843	621,643.0528	−0.0066	−0.0052
3	2,833,609.8795	621,641.8945	2,833,609.8792	621,641.8935	0.0003	0.0010
4	2,833,609.1077	621,640.8471	2,833,609.1059	621,640.8489	0.0018	−0.0018
5	2,833,608.2907	621,642.1375	2,833,608.2926	621,642.1344	−0.0019	0.0031
6	2,833,608.7893	621,641.5604	2,833,608.7905	621,641.5620	−0.0012	−0.0016

**Table 8:** Comparative analysis of the sustainability performance between the proposed method and other orthophoto acquisition techniques.

Indicator	iPhone 15 Pro + Polycam	This Method	Phase One iXM	Faro Focus S	Data Source
Energy Consumption per Task (Wh)	16	50.55	220.0	160.0	[35,36,37,38]
Carbon Emissions per Task (kg CO_2_)	0.31	0.56	2.41	8.59	[39,40,41]
Equipment Cost (10,000 CNY)	1	4.5	50.0	60.0	Manufacturer’s Official Price
Community Accessibility	High	High	Low	Medium	[42]
Public Education Potential	Supports Online Sharing	Supports Online Sharing	Limited	Limited	[43]

Note: For detailed calculation sources, refer to Appendix A.

## Data Availability

The raw data supporting the conclusions of this article will be made available by the authors on request.

## Appendix A

Detailed calculation process of Table 8 data. The comparative analysis in Table 8 was conducted through the following calculation methods:

1. Energy Consumption Calculation

Formula:

Energy (Wh)=Power (W)×Time (h)

Calculations:

LiDAR + Camera (Baseline):

4 W×4 h=16 Wh

This Method:

sony⋅A6000(15.55 Wh)+Total⋅Station(8 Wh)+LED⋅Light(27 Wh)=55.55 Wh

Phase One iXM:

55 W(rated⋅power)×4 h=220 Wh

Faro Focus S:

40 W(avg⋅power)×4 h=160 Wh

2. Carbon Emissions Calculation

Formulas:

Electricity Emissions (kg CO_2_):

$$Electricity\;(\mathrm{kWh})\times Grid\cdot Factor\;(\mathrm{kg}\times {\mathrm{co}}_{2}/\mathrm{kWh})$$

China Grid Factor = 0.581 kg CO_2_/kWh.

Embodied Emissions (kg CO_2_/task):

$$\frac{Device\cdot Production\cdot Emissions\;(\mathrm{kg}\times {\mathrm{co}}_{2})}{Lifespan\;(\mathrm{years})\times Tasks/Year}$$

Assumptions: 5-year lifespan, 52 tasks/year (1 task/week).

Total Emissions:

Electricity⋅Emissions+Embodied⋅Emissions

Calculations:

iPhone 15 Pro + Polycam:

Electricity:

$$0.022\;\mathrm{kWh}\times 0.581\;\mathrm{kg}\cdot {\mathrm{co}}_{2}/\mathrm{kWh}=0.0128\;\mathrm{kg}\cdot {\mathrm{co}}_{2}$$

Embodied:

$$\frac{79\;\mathrm{kg}\cdot {\mathrm{co}}_{2}}{5\times 52}=0.30\;\mathrm{kg}\cdot {\mathrm{co}}_{2}/\mathrm{task}$$

Total:

$$0.0128+0.30\approx 0.31\;\mathrm{kg}\cdot {\mathrm{co}}_{2}$$

This Method:

$$0.53\;\mathrm{kg}(production)+0.029\;\mathrm{kg}(electricity)=0.56\;\mathrm{kg}\cdot {\mathrm{co}}_{2}$$

Phase One iXM:

Embodied:

$$\frac{600\;\mathrm{kg}}{250\mathrm{uses}}=2.4\;\mathrm{kg}\cdot {\mathrm{co}}_{2}/\mathrm{task}$$

Electricity:

$$0.22\;\mathrm{kWh}\times 0.581=0.13\;\mathrm{kg}\cdot {\mathrm{co}}_{2}$$

Total:

$$2.4+0.13\approx 2.53\;\mathrm{kg}\cdot {\mathrm{co}}_{2}$$

Faro Focus S:

Embodied:

$$\frac{2125\;\mathrm{kg}}{250}=8.5\;\mathrm{kg}\cdot {\mathrm{co}}_{2}/\mathrm{task}$$

Electricity:

$$0.16\;\mathrm{kWh}\times 0.581=0.093\;\mathrm{kg}\cdot {\mathrm{co}}_{2}$$

Total:

$$8.5+0.093\approx 8.59\;\mathrm{kg}\cdot {\mathrm{co}}_{2}$$

3. Equipment Cost

Values:

iPhone 15 Pro + Polycam: ¥10,000 (device + subscription).

This Method:

sony⋅A6000(0.5 M)+Total⋅Station(3 M)+RTK(1 M)=4.5 million× CNY

Traditional Equipment:

Phase One iXM: ¥500,000 (with lens).

Faro Focus S: ¥600,000 (manufacturer quote).

4. Community Accessibility

This method relies on widely available equipment (smartphone assistance), while traditional techniques require specialized hardware and software.
